# Ripples of change: transforming lives and communities through sport in Colombia

**DOI:** 10.3389/fspor.2024.1504966

**Published:** 2024-11-28

**Authors:** Pedro Danilo Ponciano Núñez, Alexis Lyras, Carlos Matus-Castillo, Frano Giakoni-Ramírez, Daniel Duclos-Bastias

**Affiliations:** ^1^ Departament of Physical Education, Sport and Leisure, Universidad del Valle de Guatemala, Guatemala City, Guatemala; ^2^ School of Pharmacy and Health Professions, University of Maryland Eastern Shore, Maryland City, MD, United States; ^3^ Department of Sports Sciences and Physical Conditioning, Faculty of Education, Universidad Católica de la Santísima Concepcion, Concepcion, Chile; ^4^ Faculty of Education and Social Science, Universidad Andres Bello, Santiago, Chile; ^5^ iGEO Group, School of Physical Education, Pontificia Universidad Católica de Valparaíso, Valparaiso, Chile; ^6^ IGOID Research Group, Faculty of Sport Science, University of Castilla-La Mancha, Toledo, España

**Keywords:** life skills, civic values, youth development, sports education, sport-for-development theory, non-governmental organization

## Abstract

The Colombianitos Foundation (CF) is a pioneering youth development organization that utilizes football as a pedagogical instrument to promote life skills, civic values, and social inclusion in vulnerable communities across Colombia. This study aims to challenge the foundational assumption that sport can catalyze positive change and impart civic values, by investigating the perceived change and efficacy of the CFs Goals for a Better Life program on positive youth development in Colombia. Employing a qualitative research methodology, 28 in-depth interviews with administrators and coaches were conducted, followed by a content analysis to identify thematic patterns. The findings reveal CF's transformative capacity in cultivating human capital, social and societal capital, and distinct programmatic characteristics. CF's interventions foster significant behavioral changes, such as improved conflict resolution and non-violent communication, while promoting essential life skills and social cohesion. Moreover, the study illustrates CF's role in generating a ripple effect, wherein the benefits extend beyond individual participants to their families and the broader community. CF challenges entrenched gender norms through its mixed-gender team structure, aligning with SDG 5, and demonstrates a commitment to fostering peaceful, inclusive societies in line with SDG 16. By grounding its approach in evidence-based evaluation and leveraging strategic partnerships, CF exemplifies a sustainable model of sport-for-development. The study contributes to a deeper understanding of sport's potential to drive long-term social transformation, offering empirical insights for future research and intervention design in post-conflict and marginalized settings.

## 1 Introduction

The concept of the ideal citizen, as conceptualized in ancient Greece, was founded on a deliberate and sophisticated integration of education and physical activity, aiming to channel human aggression toward constructive and peaceful societal contributions (1). This period established a pedagogical framework that combined intellectual pursuits—art, wisdom, and technology—with physical activities, emphasizing rule adherence, mutual respect, and teamwork (2). These foundational principles were intended to shape conscientious citizens capable of contributing to the harmony and advancement of their communities. This fusion of educational and physical engagement laid the groundwork for social innovation, a philosophy that finds clear parallels in contemporary effort to achieve the Sustainable Development Goals (SDGs) (3).

The SDGs, emerging as a continuation of the Millennium Development Goals, gained global attention during a series of meetings leading up to the 2012 UN Conference on Sustainable Development in Rio de Janeiro (4). Their primary objective was to address urgent global environmental, political, and economic challenges (5). After several years of intensive work, the 17 SDGs were officially unveiled and ratified by the UN General Assembly at the 2015 Sustainable Development Summit (6). Although the SDGs are comprehensive, their specific targets are often constructed at national or global levels, which can present challenges for sectors like sports organizations to demonstrate their direct contribution to these goals. Specifically, the development of standardized metrics that allow organizations to measure and report their contributions to these goals would enhance the capacity of the sector to demonstrate its social, economic, and environmental impact. To address this, various international sport-for-development programs, such as the International Olympic Committee's “Olympic Agenda 2020”, have started incorporating mechanisms to evaluate their impact on the SDGs, showing the growing importance of sport with these global objectives (7). Nevertheless, despite its positive aspects, it is crucial to recognize that sports can also reinforce negative societal structures, such as exclusion, nationalism, and even violence in certain contexts (8–10).

However, the historical role of sport as a catalyst for social transformation—rooted in the ancient Greek tradition of fostering civic responsibility through physical activity- continues to resonate in the contemporary context of sustainable development (11). Modern sports, much like in the past, provide a platform for facilitating societal contributions by addressing pressing issues such as gender equality (SDG 5), reducing inequalities (SDG 10), and promoting responsible consumption and environmental sustainability (SDG 12 and 13). Pierre de Coubertin's vision of the modern Olympic Games, centered around education and moral development, echoes these timeless ideals by positioning sport as a conduit for fostering humanity's progress (12). This vision not only builds on ancient traditions but also aligns seamlessly with the broader ambitions of the SDGs.

In recent years, however, scholars and practitioners in Sport for Development and Peace (SDP) have questioned the limited representation of Global South voices within the field, pointing out the dominance of narratives from the Global North, which may overlook local epistemologies and culturally specific practices (13). While most SDP projects are implemented in regions such as Africa, Asia, and Latin America, a staggering 90% of SDP literature is authored by scholars based in North America, Europe, and Australia. This imbalance restricts the visibility of diverse approaches and narratives that emerge from communities where SDP initiatives are most needed and actively applied (14). Efforts to address this gap have recently gained momentum. For instance, organized an open call for Global South practitioners, researchers, and community leaders to contribute their perspectives on SDP in multiple languages, such as English, Spanish, Portuguese, and Swahili (13). Such inclusive initiatives reveal the depth and diversity of SDP practices in regions like Latin America, where local actors play a pivotal role in adapting SDP programs to address specific community needs (15). This study aligns with this evolving body of work by focusing on the Colombianitos Foundation (CF), a Colombian organization that uses sport to drive social change within post-conflict communities. By examining CF's program “Goals for a Better Life,” this research highlights the transformative potential of locally led SDP initiatives that are responsive to the unique socio-cultural and historical contexts of the Global South (13, 16).

This study aims to add to the decolonial perspectives within SDP by illustrating how CF's community-based approach aligns with the SDGs, particularly SDGs 5 and 16, which emphasize gender equality, peace, and justice. By foregrounding these local perspectives, this research supports calls to “step outside privileged positions” and validate the innovative, contextually adapted methodologies pioneered by Global South practitioners (17). Through this approach, CF demonstrates how sport, when grounded in local knowledge and cultural relevance, can serve as a powerful tool for sustainable development, empowerment, and social justice.

## 2 Football for peacebuilding

Colombia, with its enduring history of poverty and violence, particularly in football where drug traffickers have infiltrated the professional league (18, 19), serves as an example of the potential for sport to drive positive social change. The assassination of Andres Escobar following an own goal during the World Cup underscored the profound impact of football within the Colombian context and catalyzed Jurgen Griesbeck's efforts to reconceptualize the sport as a tool for conflict resolution and violence reduction (20). Griesbeck's strategic approach hinged on two critical conditions: the establishment of agreements and the fostering of communication and teamwork (21). The “Fútbol por la Paz” (FPP) initiative (1996) enshrined three foundational principles: the prohibition of firearms, the promotion of inclusive teams, and the reduction of referee intervention (21). This model is believed to have influenced the subsequent “Colombia Joven” initiative, a nationwide governmental program that adopted the moniker “Golombiao” (22). Golombiao sought to foster social inclusion in regions ravaged by conflict (23). As Non-Governmental Organizations (NGOs) such as the CF and others increasingly recognized football as a vehicle for community impact, they established partnerships with governmental stakeholders. The initiative (FPP) evolved into an official policy endorsed by sport authorities, club managers, and professional players, propelling FPP nationwide for a decade (24). Despite the proliferation of NGOs employing football as a tool for community impact, academic research on their effectiveness, particularly in the Global South, remains limited (14, 25). In Golombiao, football was used to encourage peaceful conflict resolution and build community resilience in regions devastated by years of internal violence. This study aims to challenge the foundational assumption that sport can catalyze positive change and impart civic values, by investigating the perceived impact and efficacy of the CF's “Goals for a Better Life” program on positive youth development in Colombia.

**Figure 1:**
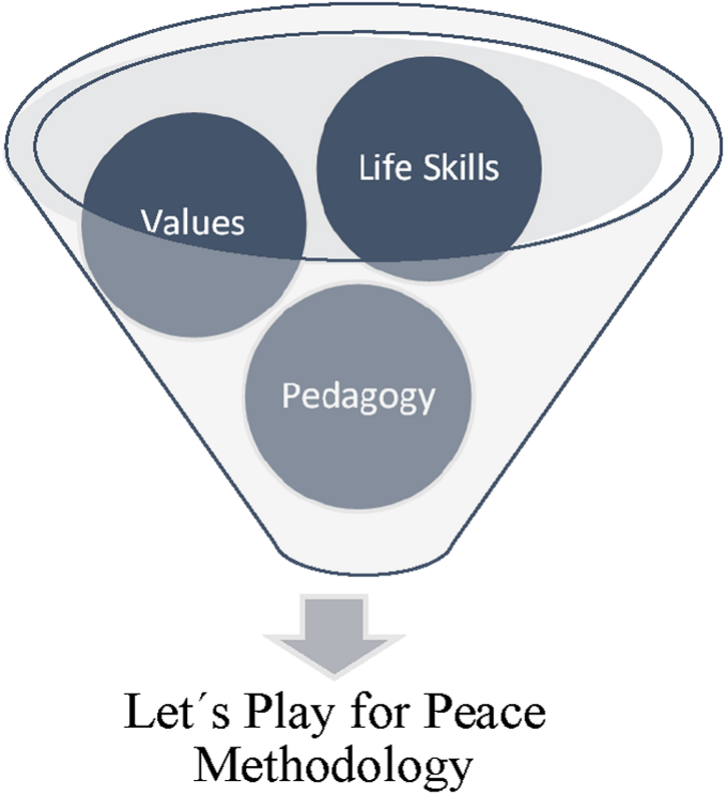
Components of Let's play for peace methodology.

The CF strategically invests in improving the quality of life for children in vulnerable communities, leveraging football as a pedagogical tool. Operating across both educational and neighborhood domains, CF's curriculum of Coexistence and Peace is utilized through the program “Goles para una Vida Mejor” (Goals for a Better Life), is grounded in the principles of FPP (26). The pedagogical model adopted by CF, “Juguemos por la Paz” (Let's Play for Peace) (Figure 1), is a curriculum designed to impart civic values and life skills, aiming to cultivate local champions capable of driving change within their communities using football as a common language (27). Figure 1 provides an overview of the curriculum, which is designed to teach psychosocial skills and civic values through football, and this infuses a holistic approach CF takes in addressing both physical and social development.

The model encapsulates the teaching of psychosocial skills, civic values, and pedagogical content, and is structured around three components adapted to the national educational curriculum for physical education: sport training with life skills education, tournaments with pedagogical intent to apply life skills, and the inclusion of non-sport educational content. The sessions are composed of four phases: the initiation phase with agreements between participants, specific sports skills training integrated with life skills development, games with pedagogical intent, and a reflective phase where participants and coaches assess the learning process.

**Figure 2:**
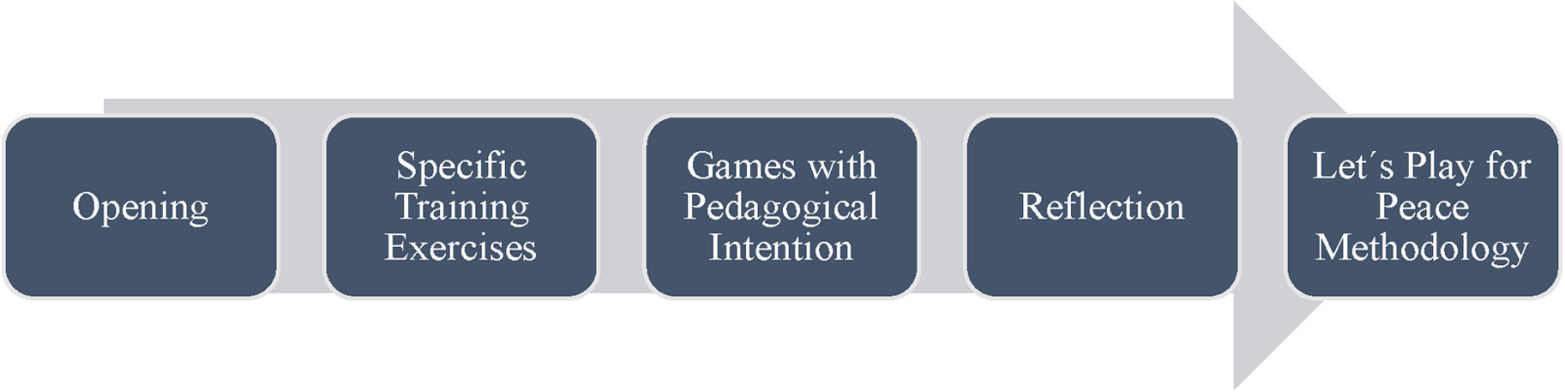
Phases of Let's play for peace methodology training session.

The sessions are structured around three components adapted to the nationwide educational system curriculum for physical education (27): sport training with life skills education, tournaments with pedagogical intentionality to apply life skills, and inclusion of other educational non-sport content. Also, encompass four phases (Figure 2): elaborate on the session structure by depicting the four phases each session follows: initiation and agreements, sports skills development integrated with life skills, games with pedagogical intent, and a reflective phase. These phases demonstrate how CF methodically combines physical activity with life skills education, fostering both individual and community development. The inclusion of life skills training in the session phases is crucial for promoting behavioral change and social cohesion, which are central to CF's mission.

## 3 Sport-for-development-theory into action

The Sport-for-Development Theory (SFDT), developed by Lyras and expanded by Lyras and Welty Peachey, offers a comprehensive framework that leverages the interdisciplinary nature of sport as a tool for social transformation (28, 29). SFDT is grounded in diverse theoretical fields, including organizational theory, psychology, and intergroup contact theory, enabling it to bridge academic research and practical field applications. In this study, SFDT serves solely as a guiding framework for evaluating CF's impact rather than for theory validation, allowing for a focused examination of how CF's sports interventions promote social change. This makes SFDT a relevant framework for sport-based interventions aimed at addressing global challenges such as peacebuilding, conflict resolution, and social inclusion, particularly aligning with Sustainable Development Goal (SDG) 16, which promotes peaceful and inclusive societies (4).

SFDT is structured around five core components: impact assessment, organizational dynamics, sport programming, educational integration, and cultural enrichment (25). Together, these elements guide the design, implementation, and evaluation of sport-for-development programs. SFDT emphasizes inclusive, adaptable programming that fosters engagement across diverse groups and enables the transfer of socio-psychological skills—like teamwork and conflict resolution—into broader societal contexts, thus supporting both individual and collective growth.

Cultural enrichment also plays a critical role, advocating for the integration of cultural activities to foster social cohesion and understanding, especially in post-conflict settings. Programs like the Doves Olympic Movement Project (DOVES), which promoted interethnic understanding between Turkish and Greek Cypriots, exemplify SFDT's applicability in peacebuilding efforts (30).

This study uses SFDT as a guiding framework to evaluate the role of sport programs, specifically exploring how CF's community-based interventions may foster personal and community growth without aiming to validate SFDT itself. Grounded in SFDT principles, this study seeks to understand how sport can function as a catalyst for positive change through its structured approach to community development.

The SFDT served as a guiding framework for this study, providing a structured approach to examine the role of sport programs in fostering community-focused development. Rather than aiming to validate the SFDT as a viable theory in practice, this study employed the SFDT principles to explore how sport-based interventions might influence personal and community growth. With SFDT as a foundation, the following research questions directed the investigation:

**RQ1:** How do administrators and coaches perceive the impact of community-focused sports programs on their own professional and personal development?
**RQ2:** What key features within community-focused sports programming contribute to optimizing positive socio-psychological outcomes, according to administrators and coaches?

## 4 Methodology

Fieldwork conducted from May to August 2018 involved pre-planning and implementation phases. Initial Skype interviews with three development and peacebuilding scholars established criteria for selecting CF. The criteria included post-conflict region location, time of existence, perceived impact, and the number of locations of CF where during that time the program was running nationwide. Subsequent mapping of CF's organizational structure, program types, and objectives was conducted. A detailed Skype interview with the CF director provided insights into each location's uniqueness and challenges. Data analysis informed methodological refinements and an in-depth content analysis.

The four-month procedure comprised two phases. Phase I involved office experience at CF headquarters and an overview of the nationwide structure, programs, and objectives. Phase II included field visits to six locations across five regions with the objective of not only observing the ideal conditions where the program is implemented but also observing the reality of its implementation in regional centers, according to the specific challenges of each context. Participants included administrative staff (*n* = 15) and coaches (*n* = 13), ranging in age from 18–45 years, with involvement in the program spanning from 2 months to 14 years, ensuring a comprehensive representation of perspectives from various levels of experience.

Methodological consistency was not only maintained by scheduling interviews but also by standardizing procedures and interviewer training. The first author was the sole interviewer, trained in neutral interviewing techniques and following a semi-structured guide to enhance reliability across sessions. This study followed the Declaration of Helsinki (Hong Kong, September 1989) (31) and was reinforced by the Bangkok Declaration on Physical Activity for Global Health and Sustainable Development (32).

### 4.1 Personal interviews

Semi-structured interviews served as the primary method for gathering insights from coaches and administrative staff involved in the program's design, implementation, and evaluation. This approach allows for an in-depth exploration of participants' perceptions and beliefs (33, 34). Questions were tailored to each participant's role within the organization, fostering a comfortable environment that encouraged open expression (38, 40, 41). The interview guide (Table 1) was based on concepts derived from the SFDT framework, as depicted in Table 1. The demographic breakdown of participants is shown in Tables 2, 3.

**Table 1:** Dimensions of the semi-structured interview.

Dimension	Description	Coaches	Administrative Staff
Management structures and practices	Questions focused on the administrative model of CF, their organizational structures, processes, and sustainability	✔	✔
Business model and characteristics	Details on the business model, distinctive organizational characteristics, and success factors		✔
Threats and challenges	Questions regarding challenges in the design, implementation, and sustainability of programs	✔	✔
Perceived impact	Questions about the perceived impact of the foundations on participants and the community	✔	✔
Role of sports in programming	Questions related to the role of sports in achieving the foundations' objectives	✔	
Educational and cultural components	Questions about the use of educational topics, pedagogy, and cultural enrichment components		✔

The average interview length was approximately 30 min, exceeding the initially planned 20 min. This provided sufficient time to probe participants' insights more thoroughly, reaching data saturation as recurring themes emerged. Data saturation was monitored by analyzing transcripts continuously until no new information was found.

### 4.2 Data analysis

Interviews, ranging from 20–60 min, were recorded, transcribed, and translated from Spanish to English in their entirety. The participants included administrative staff (*n* = 15, 4 men, 11 women) (Table 2) and coaches (*n* = 13, 9 men, 4 women) (Table 3) involved in the youth program. The thematic and axial coding of the transcripts followed a deductive approach, aligning with priori themes and concepts grounded in validated scales of the SFDT (35, 36). The broader impact of CF's interventions is demonstrated through the “Ripple Effect” as illustrated in Table 3.

**Table 2:** Demographic information CF administrative staff.

Code	Position	Profession	Gender
PA1	National Manager	Lawyer	F
PA2	Educative assessor	Pedagogue	F
PA3	Operational director	Anthropologist	F
PA4	Operational director	Sport professional	M
PA5	Regional administrator	Social worker	F
PA6	Regional administrator	Sport professional	M
PA7	Regional administrator	Sport professional	M
PA8	Regional administrator	Psychologist	F
PA9	Regional administrator	Sociologist	F
PA10	Regional administrator	Pedagogue	F
PA11	Regional administrator	Psychologist	F
PA12	Regional administrator	Social worker	F
PA13	Regional administrator	Social worker	M
PA14	Regional administrator	Primary school teacher	F
PA15	Regional administrator	Pedagogue	F

**Table 3:** Demographic information CF coaches.

Code	Position	Profession	Gender
PC1	Coach	Sport professional	M
PC2	Coach	Sport professional	M
PC3	Coach	Sport professional	F
PC4	Coach	Sport professional	M
PC5	Coach	Sport professional	M
PC6	Coach	Physical education teacher	F
PC7	Coach	Soccer coach	M
PC8	Coach	Soccer coach	M
PC9	Coach	Soccer coach	F
PC10	Coach	Sport professional	M
PC11	Coach	Social worker	F
PC12	Coach	Sport professional	M
PC13	Coach	Sport professional	M

The transcripts were encoded using Atlas.ti 20, and response frequencies were analyzed to identify recurring themes. To ensure confidentiality, a code was provided to the participant during transcription and coding. As the first author of this study, We acknowledge our positionality as a non-Colombian researcher examining a context deeply rooted in the country’s cultural and historical fabric. Since I was solely responsible for data collection and analysis, a positionality statement has been included to address potential bias. This was balanced by regular discussions with co-authors to validate interpretations and achieve a broader perspective on the findings.

Recognizing that only CF program staff and administrators were interviewed, this study specifically reflects their perspective on the program's impact. We acknowledge this as a limitation and note that direct input from participants and the broader community was not obtained, which will be addressed in the limitations section. While our background in sport-for-development provides a valuable external perspective, We recognize that my outsider status may have influenced both the interpretation of the data and my interactions with participants. This distance, however, allowed me to approach the research with a level of objectivity, offering insights that may not be as readily visible to those embedded within the local context. To mitigate potential limitations in cultural understanding, I was conscious of building trust with participants and local stakeholders, particularly given the sensitive nature of the post-conflict environment in Colombia. This was facilitated through prolonged engagement and the use of culturally informed methodologies. My position as an outsider not only posed challenges but also enriched the study by allowing for cross-cultural comparisons and global contextualization of the findings.

The primary aim of this study was to explore the perceived impact of the CF program on both individual and community levels, as viewed by administrative staff and coaches. Specifically, the study sought to understand how the program's interventions contribute to personal development, social cohesion, and sustainable community outcomes within post-conflict environments.

**Figure 3:**
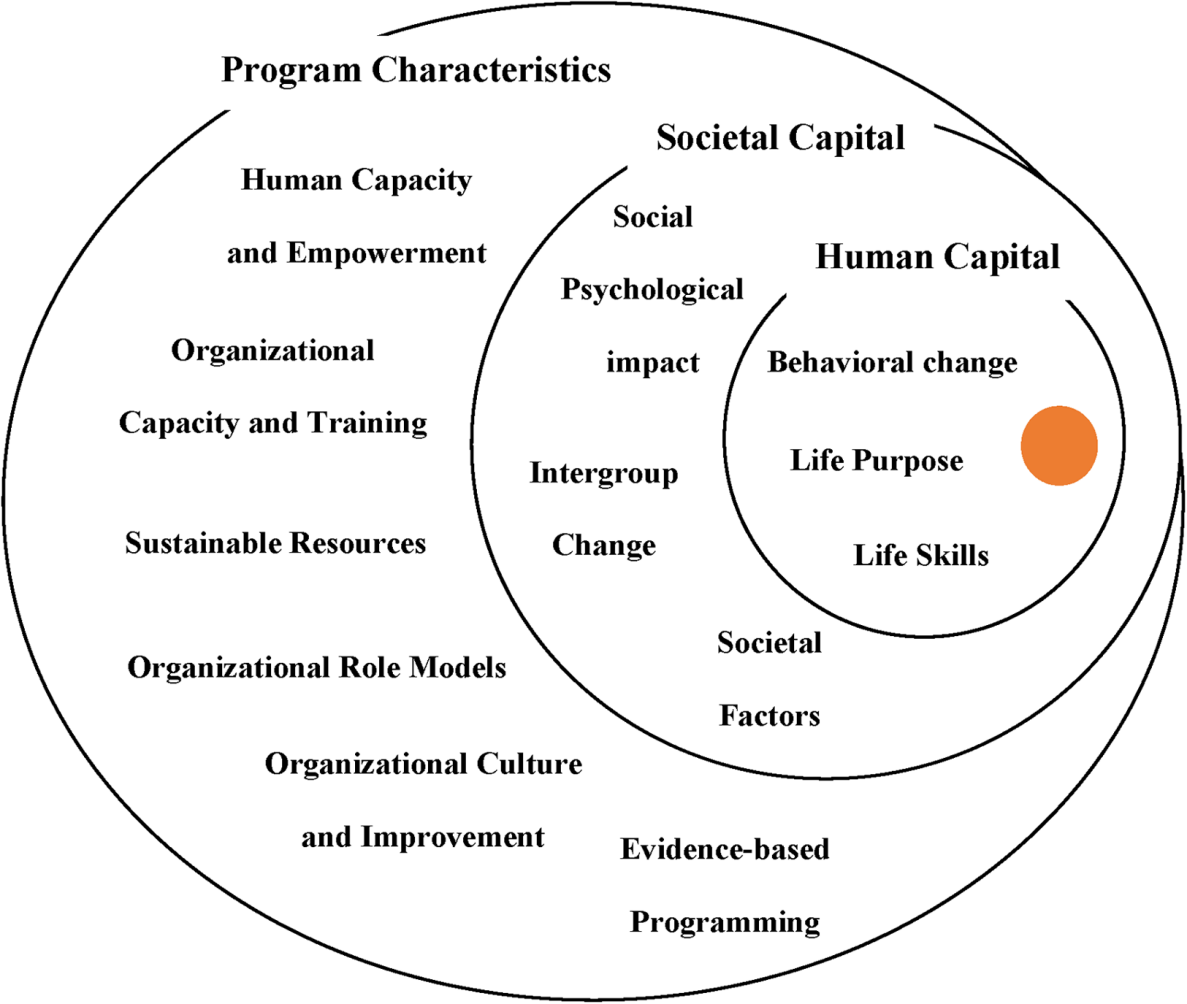
Ripple effect based on SFDT.

The findings are presented through three main themes that emerged from the analysis of the interviews: Human Capital, Societal Capital, and Program Characteristics. Each of these themes is composed of several subthemes that detail the different dimensions of CF's program impact. These themes and subthemes are visualized in Figure 3, which shows how the program's interventions generate a Ripple Effect that extends from individual participants to their communities. The figure highlights the relationships between the main themes and their subthemes: Human Capital includes the subthemes of behavioral change, life purpose, and life skills; Societal Capital reflects the psychosocial impact, intergroup changes, and societal factors; while Program Characteristics encompasses human capacity development, organizational training, and sustainable resources. This visual representation in Figure 3 helps illustrate how the themes and subthemes are interconnected and contribute to the overall impact of the program.

## 5 Findings

The primary aim of this study was to explore the perceived impact of the CF program on both individual and community levels, as viewed by administrative staff and coaches. Specifically, the study sought to understand how the program's interventions contribute to personal development, social cohesion, and sustainable community outcomes within post-conflict environments.

The findings are presented through three main themes that emerged from the analysis of the interviews: Human Capital, Societal Capital, and Program Characteristics. Each of these themes is composed of several subthemes that detail the different dimensions of CF's program impact. These themes and subthemes are visualized in Figure 3, which shows how the program's interventions generate a Ripple Effect that extends from individual participants to their communities. The figure highlights the relationships between the main themes and their subthemes: Human Capital includes the subthemes of behavioral change, life purpose, and life skills; Societal Capital reflects the psychosocial impact, intergroup changes, and societal factors; while Program Characteristics encompasses human capacity development, organizational training, and sustainable resources. This visual representation in Figure 3 helps illustrate how the themes and subthemes are interconnected and contribute to the overall impact of the program.

### 5.1 Human capital

The first theme pertains to the individual impact of CF's programming within communities affected by violence and conflict. The program significantly contributes to the personal development of participants, as evidenced by three key subthemes: (*i) behavioral change, (ii) life purpose, and (iii) life skills*.

#### 5.1.1 Behavioral change

CF's programming has been instrumental in promoting behavioral changes among participants and enhancing their conflict resolution skills. The integration of pedagogical components within sport practice aims to foster behavioral changes such as respect, discipline, and non-violent problem-solving methods, which starkly contrast with the typical responses ingrained in the local culture.

As PA1 noted: “Children have been with us for three years now, and what I hope is that they will eventually reject violence as an option—a behavior so prevalent in our region—as they pursue their life goals.”

This observation underscores the hope and expectation that participants will gradually internalize new behaviors over time, moving away from culturally ingrained norms of violence. While the desired behavior change is not guaranteed, CF's approach is consistent with behavior change models, which emphasize the role of positive role models and continuous educational intervention in promoting lasting behavioral change (37, 38). Behavioral change remains an important indicator of the program's potential impact, suggesting that participants are gradually developing new ways of interacting with their environment.

PA4 further highlighted: “You can see the empathy and values that emerge from CF's methodological approach. Where once a misunderstanding on the pitch would end the game abruptly, now participants calm down, discuss the issue, and apologize.”

This quote illustrates shifts in social dynamics among participants, indicating an early internalization of social skills such as empathy and conflict resolution. Although CF aims to instill these skills in participants, the process is ongoing and reflects a gradual cultural shift within communities, contributing to the accumulation of social capital (39, 40). This phenomenon aligns with theories that posit individuals learn behaviors by observing and imitating others, particularly in structured environments supported by educators and coaches (37, 38). The ability of participants to de-escalate conflicts and engage in constructive dialogue suggests that the program is progressively instilling collaborative problem-solving skills.

#### 5.1.2 Life purpose

CF's methodology also influences participants' decision-making, guiding them to develop clear life projects that benefit both themselves and their communities. However, it is important to recognize that the perspectives shared here reflect the views of CF program administrators and coaches, who may hold a natural optimism and pride in their work, potentially leading to a positive bias in their assessments.

As PA6 shared: “I am proud that our heartfelt work has yielded visible results—life stories of young people who, despite their circumstances, have become sources of pride for us and their families thanks to CF.”

This statement reflects the personal investment and pride that administrators feel in the transformative potential of CF. While this perspective suggests that some participants may experience deep personal growth, it is acknowledged that these observations are based on administrators' perceptions rather than direct reports from participants. This aligns with theories of positive youth development, which emphasize the importance of fostering a sense of direction and meaning (41). The pride expressed by PA6 underscores the program's aspiration to cultivate intrinsic motivation and resilience among participants, although further research involving participant voices would provide a more balanced view.

Similarly, PC1 noted that despite their vulnerability, children in the program are beginning to aspire to better life opportunities: “There are many impacts, but the first is that children want a life project. They want to do something positive; they don't want to remain in the same difficult environment.”

This quote highlights the program administrators' perception that CF instills a shift in participants' aspirations and mindset. However, given that these insights come from staff members, there is a recognized need for future studies to incorporate direct participant feedback to confirm and expand on these observations. The desire to pursue a life project, as perceived by the administrators, suggests a shift toward growth and improvement. This aligns with the concept of “future orientation,” which is critical for breaking cycles of poverty and violence (42).

#### 5.1.3 Life skills

The programme's methodology also fosters a respectful and collaborative classroom environment, reducing aggression and providing a space for participants to acquire vital life skills.

PA7 noted: “*The implementation of CF's methodology has been incredibly positive. We've seen changes in students who were initially reluctant to participate. The innovative, reflective, and participatory activities have been pivotal in teaching life skills and citizenship.”*

PA7's observation emphasizes the effectiveness of CF's innovative pedagogical approach, which not only engages students but also facilitates the development of critical life skills. The reflective and participatory nature of the activities aligns with experiential learning theories, which suggest that active involvement in learning processes leads to deeper understanding and retention of skills (43). The emphasis on citizenship indicates that the program is also successful in instilling a sense of social responsibility among participants, preparing them to be constructive members of their communities.

PA12 further highlighted that CF is known for imparting life skills through sport, integrating psychosocial and pedagogical elements: “*CF is renowned for teaching life skills through sport. In Manizales, we had an advantage by combining psychosocial and pedagogical aspects, which participants later applied in their lives.”*

This statement underscores the holistic approach of CF, which goes beyond traditional sports education to include psychosocial support and pedagogical training. The integration of these elements reflects an understanding of the multifaceted nature of development, where cognitive, emotional, and social skills are interconnected (44). The ability of participants to apply these skills in their daily lives suggests that the programme's impact extends beyond the immediate context of sports, influencing broader aspects of their personal development and social interactions.

### 5.2 Societal capital

The second theme captures the broader community impact of CF's programming. This includes the psychological benefits for those close to the participants, the overcoming of stereotypes and prejudices, and the programme's effect on the wider community. The subthemes identified were: *(i) social-psychological impact, (ii) intergroup change, and (iii) societal factors*.

#### 5.2.1 Social-psychological impact

Coaches and trainers, closely involved with participants, experience a profound impact, gaining heightened social sensitivity as they observe how sport can bring together youth from conflicted backgrounds.

PC2 expressed: “The impact is unbelievably valuable. I would describe it as beautiful—seeing groups who previously did not speak, now playing together peacefully, celebrating goals. It's a genuine rescue of values and an example of inclusive education.”

PC2's statement highlights the transformative power of sport as a medium for social integration. The description of the impact as “beautiful” underscores the emotional resonance of witnessing previously divided groups coming together. This observation aligns with Intergroup Contact Theory, which posits that under certain conditions, intergroup contact can reduce prejudice and foster positive relationships (45).

For intergroup contact to be effective, several conditions are typically required: equal status among participants, common goals, cooperative interaction, and institutional support. CF's programming meets these conditions by creating an environment where participants from various backgrounds work together on equal footing, with a shared goal of teamwork and sportsmanship. Additionally, CF's commitment to inclusive and supportive programming provides the necessary institutional backing, which is crucial for reducing social tensions. The notion of “rescuing values” suggests that CF not only brings individuals together but also fosters a reclamation of communal harmony and social cohesion, bridging divides within the community.

PC5, a former participant who became a coach, shared how CF changed his life: “I come from a neighborhood plagued by drug addiction and poverty. Through CF, I found a way to spend my free time in sports, which became a tool for transmitting values. This experience led me to become a football coach in my community.”

PC5's journey from participant to coach illustrates the program's long-term impact on individual trajectories. His transformation from a vulnerable youth to a community leader exemplifies the Ripple Effect of CF's interventions, where initial participants become agents of change in their own communities. This aligns with the concept of “empowerment,” where individuals gain control over their lives and the capacity to effect social change (46). His ability to internalize and later disseminate the values learned through CF reflects the program's success in creating sustainable change through a cascading model of influence.

#### 5.2.2 Intergroup change

CF's methodology encourages intergroup contact by promoting mixed-gender teams, which fosters coexistence, empowerment, confidence, and conflict resolution skills. By encouraging both men and women to participate on equal footing, CF creates a space where traditional gender roles are re-evaluated, promoting respect and mutual understanding.

PA13 noted: “Women play a crucial role as equal participants in football, not just with their gender but alongside men. This has a significant impact on our participants.”

PA13's comment highlights the role of CF in challenging and redefining gender norms within the community. The “significant impact” mentioned by PA13 refers to shifts in participants' perceptions regarding gender roles, where both male and female participants begin to see each other as equals on the field. Observing women actively participate and compete fosters an environment that challenges existing stereotypes about gender roles in sport and society at large. Male participants, for instance, may come to recognize women as capable teammates rather than competitors or outsiders, while female participants gain a sense of empowerment and confidence in traditionally male-dominated spaces. This process aligns with feminist theories of empowerment, which argue that creating spaces for women in areas traditionally dominated by men is essential for achieving gender equality (47). The impact of these mixed-gender interactions is not only evident in the way participants play but also in the relationships they form, which reflect greater inclusivity and shared respect.

PA6 added that the inclusion of women is fundamental to the success of the methodology: “Women are essential. By working on inclusion and equity, they feel deeply involved and empowered, fully engaging with the activities and processes.”

PA6's statement reinforces the importance of gender inclusion as a cornerstone of CF's methodology. Female participants who are engaged in mixed-gender teams experience empowerment by being treated as equals, which enhances their confidence and willingness to actively participate. This sense of inclusion fosters an environment where women feel valued, which is crucial for their sustained engagement and personal growth. The emphasis on equity and active participation reflects CF's commitment to creating an environment where all participants, regardless of gender, are valued and empowered. This approach aligns with inclusive education principles, which advocate for the active participation of all students in learning processes, particularly those from marginalized groups (48). The empowerment of female participants through these interactions suggests that CF's interventions are contributing to the long-term goal of gender equality and reshaping social norms within the community.

#### 5.2.3 Societal factors

The programme's influence extends beyond the participants, impacting their families and encouraging non-violent problem-solving.

PA15 shared: “*The impact reaches beyond the child to the parents. After the project in Manizales, hearing parents talk about how they've stopped tolerating certain types of aggression shows that our work has truly changed lives.”*

PA15's observation highlights the ripple effect of CF's interventions, where the impact on individual participants extends to their families and wider community. The shift in parental attitudes towards aggression suggests that the program is fostering a broader cultural change, where non-violent behaviors are becoming normative. This observation aligns with ecological systems theory, which posits that changes within an individual's immediate environment (microsystem) can influence larger societal structures (macrosystem) (35, 44). The programme's success in altering family dynamics indicates its potential to drive long-term social change by reshaping community norms and values.

PA13 emphasized the importance of teachers and coaches in applying CF's methodology with diverse groups, highlighting the need for personal impact to effectively motivate others: “*If I don't feel the impact myself, I can't impact others. I must not only learn the methodology but experience it to inspire participants in schools or neighborhoods.”*

PA13's statement underscores the importance of personal transformation as a precursor to effective leadership and influence. This aligns with transformational leadership theory, which suggests that leaders who are deeply committed to and transformed by their own experiences are more effective in inspiring and motivating others (49). The emphasis on experiential learning reflects the programme's holistic approach, where educators and coaches are not merely transmitters of knowledge but active participants in the learning process. This ensures that the methodology is not only understood intellectually but is also internalized and authentically conveyed to participants.

### 5.3 Program characteristics

The third theme focuses on the characteristics of CF's programming, which serves as a catalyst for positive impact across time and space. The programme's structure is crucial for enabling stakeholders to achieve human development indicators, such as academic improvement and life skills. The subthemes identified include: (*i) human capacity and empowerment, (ii) organizational capacity and training, (iii) sustainable resources, (iv) organizational role models, (v) organizational culture and improvement, and (vi) evidence-based programming*.

#### 5.3.1 Human capacity and empowerment

CF's commitment to enhancing academic performance and life skills as pathways to improved quality of life is central to its program structure and mission. However, it is important to recognize that the insights provided by administrators and coaches primarily reflect their expectations and the intended impact of CF's programming, rather than direct evidence of outcomes among participants.

PA11 stated: “Our goal is twofold: academic improvement and equipping children with the life skills needed to navigate the adversities they face daily.”

PA11's statement highlights CF's strategic focus on holistic development, recognizing the interconnectedness of academic achievement and life skills in improving overall quality of life. While this approach aligns with human capital theory, which posits that investments in education and skill development lead to enhanced productivity and societal well-being (50), it should be noted that PA11's comments reflect program objectives rather than documented participant outcomes. CF's goal is to position participants for success not only in educational settings but also within their broader social and economic contexts; however, future research would be necessary to confirm if these goals are being fully realized.

PC12 added that CF's unique approach lies in infusing values and life purpose through role models: “CF's vision is to provide role models that inspire participants to develop life projects. Our educational model helps build the foundation for these projects, fostering a clear vision for the future.”

PC12's comment underscores the importance placed on role models within CF's programming. The emphasis on “life projects” suggests an aspirational framework aimed at shaping participants' long-term life trajectories. This aligns with positive youth development frameworks that stress the value of mentorship in guiding young people toward fulfilling their potential (51). Nevertheless, these statements reflect the intended purpose of the program rather than direct evidence of participant outcomes. Further participant-focused studies would provide valuable insight into the actual realization of these life projects and the extent to which CF's objectives translate into tangible changes in participants' lives.

#### 5.3.2 Organizational capacity and training

PC7 highlighted CF's distinctive approach to promoting civic values and life skills, setting them apart from other organizations: “*We don't just teach values; we focus on life skills. The World Health Organization recognizes 10 life skills, and we see them as essential tools to combat the social challenges our participants face.”*

PC7's statement reflects an understanding of the importance of life skills in addressing broader social challenges. By aligning their programming with recognized frameworks such as the WHO's life skills model, CF is ensuring that their interventions are evidence-based and relevant to the needs of their participants. This approach underscores the importance of building organizational capacity to deliver comprehensive and impactful programs. The emphasis on life skills as a tool for social change aligns with theories of social capital, which suggest that equipping individuals with these skills can lead to improved social outcomes and community cohesion (52).

PC13 reiterated the importance of using sport to develop life skills, especially for children in vulnerable social environments: “*We offer free sport to children, teaching them values and life skills, helping them become better people in the face of the challenging environments they come from.”*

PC13's comment reinforces the role of sport as a vehicle for social change. The focus on providing free sport to vulnerable children indicates a commitment to accessibility and inclusion, ensuring that all participants, regardless of their socio-economic background, can benefit from the program. This aligns with the principles of equity and social justice, which are central to the mission of many sport-for-development initiatives (53). The statement also highlights the transformative potential of sport, not just as a physical activity but as a means of instilling values and life skills that can help participants navigate and overcome their challenging environments.

#### 5.3.3 Sustainable resources

Participants recognized the necessity of sustaining CF's programming through strategic partnerships, securing both national and international resources.

PA11 noted: “CF's board of directors has successfully established relationships not only with Colombian organizations but also with international entities, earning local and global recognition.”

PA11's statement highlights the importance of strategic partnerships in ensuring the sustainability of CF's programming. By building relationships with both local and international entities, CF gains access to a broader range of resources and support, which is crucial for the long-term viability of their initiatives. This aligns with resource dependency theory, which suggests that organizations must effectively manage their external dependencies to secure the resources needed for survival and growth (54).

While PA11 mentions local and global recognition, further evidence would be needed to fully understand the depth of trust and credibility associated with these partnerships. The partnerships themselves indicate that CF has established connections within the sport-for-development sector; however, future research could explore how these relationships influence CF's reputation and impact on a broader scale.

PA1 added that these connections enable CF to attract those who believe in the power of sport as a tool for social capital and educational development: “The president has connected with individuals who are passionate about sport, social change, and educational investment. This has been crucial for promoting sport at the national level as a key societal development factor.”

PA1's comment further underscores the importance of leadership in building and maintaining these strategic partnerships. The ability to connect with individuals and organizations aligned with CF's mission is essential for mobilizing the resources and support needed to advance CF's goals. This reflects the role of social entrepreneurship in the sport-for-development sector, where leaders must navigate complex networks of stakeholders to drive social change (55). The focus on sport as a key factor in societal development highlights the broader impact of CF's programming, positioning it as a potentially integral part of national development strategies.

#### 5.3.4 Organizational role models

CF's approach to developing young change agents who can serve as role models within their communities was highly praised.

PA5 shared: “*We focused on empowering young people to become multipliers of the methodology and process they experienced. This belief in their ability to replicate and sustain the process within their communities is crucial to our success.”*

PA5's statement reflects CF's strategic focus on sustainability through the development of local champions. By empowering young participants to become role models and multipliers of the programme's methodology, CF is ensuring that the impact of their interventions is both deep and lasting. This approach aligns with theories of capacity building, which emphasize the importance of developing local leadership to sustain and expand the impact of development initiatives (56). The belief in participants' ability to replicate and sustain the process within their communities suggests that CF's approach is grounded in a deep trust in the potential of young people to drive positive change.

PC8 added that CF's philosophy of using sport to shape lives has had a profound impact on his personal growth and decision to become a coach: “*At CF, I learned that football was more than just a game; it was a tool for transmitting values. This realization led me to become a coach, investing in children so they can make better decisions and move forward*.”

PC8's journey from participant to coach illustrates the transformative potential of CF's programming. The realization that sport can be a tool for transmitting values reflects a shift in perception that is crucial for the sustainability of the programme's impact. By internalizing the values taught through CF and deciding to become a coach, PC8 embodies the programme's success in creating a ripple effect, where initial beneficiaries of the program go on to become leaders and change agents within their own communities. This aligns with the concept of transformational leadership, where leaders inspire and motivate others to achieve more than they thought possible (57).

#### 5.3.5 Organizational culture and improvement

Participants emphasized the importance of CF's caring and cohesive organizational culture, which promotes a collective environment where participants feel supported in achieving their goals.

PA11 shared: “*CF provides spaces where participants can improve their minds, set goals, and receive support with their homework, creating a reference point for their development.”*

PA11's statement highlights the role of organizational culture in supporting participant development. By creating a supportive and nurturing environment, CF is fostering a culture of care that is essential for the holistic development of participants. This approach aligns with theories of organizational culture that emphasize the importance of shared values, beliefs, and practices in shaping the behavior and outcomes of organizational members (35). The provision of spaces for academic support and personal development reflects CF's commitment to addressing the multifaceted needs of its participants, ensuring that they are equipped to achieve their full potential.

PA15 added that CF invests in continuous training for teachers and improving pedagogical materials: *“Teachers are trained, supported, and provided with all necessary materials so they can continue the work independently, ensuring the programme's sustainability within educational institutions.”*

PA15's comment underscores the importance of capacity building within CF's organizational culture. By investing in continuous training and providing pedagogical materials, CF is ensuring that its educational model can be effectively implemented and sustained within educational institutions. This approach aligns with best practices in professional development, which emphasize the importance of ongoing support and resources for educators to ensure the successful implementation of new methodologies. The focus on sustainability suggests that CF is committed to building a resilient and adaptable organization that can continue to deliver high-quality programming over the long term.

#### 5.3.6 Evidence-based programming

CF's emphasis on systematic evaluation and presenting results to sponsors was highlighted as a key factor in maintaining high-quality interventions.

PA7 noted: “*We use the Yarpet Test as a baseline for evaluating participant behavior, applying it at the start and end of projects. The results are then presented to our sponsors.”*

PA7's statement reflects CF's commitment to evidence-based programming, where systematic evaluation is used to measure the effectiveness of interventions and inform future practice. The use of the Yarpet Test as a baseline tool suggests a rigorous approach to monitoring and evaluation, ensuring that the programme's impact is accurately accessed and communicated to stakeholders. This aligns with the principles of evidence-based practice, which emphasize the importance of using empirical data to guide decision-making and improve outcomes (58). The ability to present results to sponsors also highlights the role of accountability in securing continued support and resources for the program.

PA12 stressed the importance of systematic evaluation in securing resources and sustaining the project: “*To secure funding, we must systematically evaluate our work, using indicators to show sponsors the impact—whether positive or negative—and establish new commitments for continued support.”*

PA12's comment underscores the critical role of evaluation in the sustainability of CF's programming. By systematically assessing the impact of their interventions and communicating these findings to sponsors, CF can build trust and secure the resources needed to continue their work. This approach reflects the importance of accountability in development practice, where transparency and evidence of impact are key to maintaining the confidence and support of stakeholders. The focus on establishing new commitments suggests that CF is proactive in seeking opportunities to expand and deepen their impact, ensuring the long-term viability of their programming.

## 6 Conclusion

The study on CF demonstrates the potential of sports-based interventions to foster positive social change within vulnerable communities affected by violence and poverty. Using SFDT solely as a framework for evaluation, this study highlights significant behavioral shifts in participants, such as improvements in conflict resolution, discipline, and non-violent communication (42, 43). While these shifts are still developing, program administrators have noted a ripple effect that they hope will extend beyond individual participants, influencing families and communities to embrace peaceful and inclusive social norms (49). This expands the SFD literature by offering empirical data on the impact of interventions in a specific Latin American context, addressing the need to document Global South cases in a field predominantly oriented toward the Global North.

Unlike studies that document programmatic evaluations of SFD in more generalized contexts, this research explores the specific experiences and perceptions of participants and the community, capturing cultural and social dynamics that enrich SFDT. The implementation of mixed-gender teams in CF challenges traditional gender norms and promotes social inclusion, aligning with Sustainable Development Goals (SDG) 5 and 16. This culturally adapted approach not only validates the relevance of SFDT in development contexts but also suggests that SFD programs can fulfill a transformative and sustainable role in social change when designed and implemented with local realities in mind.

One of the program's anticipated outcomes is the advancement of gender equity and empowerment of women, which aligns with SDG 5. Through mixed-gender teams and by challenging traditional gender roles, CF seeks to foster inclusivity and create leadership opportunities for women within their communities, demonstrating how sport can serve as a powerful tool for social change (51, 52). Additionally, the study highlights CF's emphasis on long-term sustainability through strategic partnerships with national and international organizations. These collaborations are essential for securing resources and validating CF's approach, which contributes to the ongoing success and expansion of its programs (58).

Despite these promising aspects, several limitations of the study should be acknowledged. The small sample size limits the generalizability of the findings. While the sample was carefully selected to represent diverse roles and experiences within CF, future research would benefit from a larger and more varied participant base to enhance data representativeness. Additionally, the qualitative nature of the study introduces subjectivity, as data reflects the perspectives of program administrators and coaches, whose enthusiasm for CF's mission may shape their responses. While rigorous coding and reflexive practices were employed, further research using mixed-method approaches could triangulate findings and increase the robustness of the analysis.

Although CF has over 21 years of experience and operates in multiple regions across Colombia, understanding how these programs maintain their effectiveness over time and can be replicated in other contexts is essential for assessing their full potential. Future research should focus on longitudinal studies, especially those that capture the evolution of community dynamics and individual perceptions, to evaluate the long-term impact of CF's programs and their adaptability in diverse cultural or geographical settings. Such studies could also explore how CF's model might be tailored to different post-conflict or low-resource environments to enhance its contributions to SFDT. Additionally, examining the role of local stakeholders in sustaining these initiatives would provide valuable insights into the best practices for sports-based interventions.

These efforts align with SDG 16, which emphasizes creating peaceful and inclusive societies (5, 59). A key factor in CF's ongoing success is its commitment to evidence-based evaluation. By using tools such as the Yarpet Test to assess behavioral change, CF can continuously refine its interventions to better meet the evolving needs of the community (57, 60). Through its alignment with SFDT as an evaluative framework and the Sustainable Development Goals, particularly SDGs 5 and 16, CF illustrates how sport can serve as a catalyst for peace, inclusion, and social justice. This model offers valuable insights for future research and practice, underscoring sport's potential to contribute to long-term societal transformation in diverse global contexts (36, 61).

While this study does not seek to validate SFDT, it applies SFDT principles as an evaluative framework, offering empirical insights into the impact of sports-based interventions in a post-conflict Latin American context. The focus on gender equity and inclusion through mixed-gender teams challenges traditional gender norms, emphasizing sport's role in advancing SDGs 5 and 16. By integrating educational and cultural elements into the program structure, this study also highlights the importance of holistic approaches that support both individual and community development.

## Data Availability

The datasets presented in this article are not readily available because The data is available only under justified request to the first author. Requests to access the datasets should be directed to pdponciano@uvg.edu.gt.
