# Corrigendum: Ripples of change: transforming lives and communities through sport in Colombia

**DOI:** 10.3389/fspor.2024.1536433

**Published:** 2024-12-19

**Authors:** Pedro Danilo Ponciano Núñez, Alexis Lyras, Carlos Matus-Castillo, Frano Giakoni-Ramírez, Daniel Duclos-Bastias

**Affiliations:** ^1^ Departament of Physical Education, Sport and Leisure, Universidad del Valle de Guatemala, Guatemala City, Guatemala; ^2^ School of Pharmacy and Health Professions, University of Maryland Eastern Shore, Princess Anne, MD, United States; ^3^ Department of Sports Sciences and Physical Conditioning, Faculty of Education, Universidad Católica de la Santísima Concepcion, Concepcion, Chile; ^4^ Faculty of Education and Social Science, Universidad Andres Bello, Santiago, Chile; ^5^ iGEO Group, School of Physical Education, Pontificia Universidad Católica de Valparaíso, Valparaiso, Chile; ^6^ IGOID Research Group, Faculty of Sport Science, University of Castilla-La Mancha, Toledo, España

**Keywords:** life skills, civic values, youth development, sports education, sport-for-Development theory, non-governmental organization

A Corrigendum on Ripples of change: transforming lives and communities through sport in Colombia By Ponciano Núñez PD, Matus-Castillo C, Giakoni-Ramírez F and Duclos-Bastias D (2024). Front. Sports Act. Living. 6:1504966. doi: 10.3389/fspor.2024.1504966

Error in Author List

In the published article, there was an error in the author list, and author Alexis Lyras was erroneously [excluded]. The corrected author list appears below.

Pedro Danilo Ponciano Núñez^1^*, Alexis Lyras^2^, Carlos Matus-Castillo^3^, Frano Giakoni-Ramírez^4^ and Daniel Duclos-Bastias^5,6^

The authors apologize for this error and state that this does not change the scientific conclusions of the article in any way. The original article has been updated.

Text Correction

In the published article, there was an error. [Incorrect labeling of a section title].

A correction has been made to **[5. Methodology]**. This sentence previously stated:

“[5. Methodology]”

The corrected sentence appears below:

“[5. Findings]”

In the published article, there was an error. [Page 5].

A correction has been made to **[Data Analysis]**. This sentence previously stated:

“[I acknowledge my positionality as a non-Colombian researcher examining a context deeply rooted in the country's cultural and historical fabric.]”

“[While my background in sport-for-development provides a valuable external perspective, I recognize that my outsider status may have influenced both the interpretation of the data and my interactions with participants.]”

The corrected sentence appears below:

“[We acknowledge our positionality as a non-Colombian researcher examining a context deeply rooted in the country's cultural and historical fabric.]”

“[While our background in sport-for-development provides a valuable external perspective, We recognize that my outsider status may have influenced both the interpretation of the data and my interactions with participants.]”

In the published article, there was an error. [Acknowledgments].

A correction has been made to **[10. Acknowledgments]**. This sentence previously stated:

“[The authors (s) thank the coaches and administrative staff of CF for their involvement in this project. Also, to Prof. Alexis Lyras, for guiding this research study during the first author's master's degree and providing feedback for the last two years to improve its quality.]”

The corrected sentence appears below:

“[The authors thank the coaches and administrative staff of CF for their invaluable involvement in this project. Additionally, the authors acknowledge the support and contributions of TIAS (Tsukuba International Academy for Sport Studies) and the Sport for Tomorrow program, which provided essential guidance and resources for this research.]”

The authors apologize for this error and state that this does not change the scientific conclusions of the article in any way. The original article has been updated.

